# Health-related qualify of life, angina type and coronary artery disease in patients with stable chest pain

**DOI:** 10.1186/s12955-020-01312-4

**Published:** 2020-05-14

**Authors:** Nina Rieckmann, Konrad Neumann, Sarah Feger, Paolo Ibes, Adriane Napp, Daniel Preuß, Henryk Dreger, Gudrun Feuchtner, Fabian Plank, Vojtěch Suchánek, Josef Veselka, Thomas Engstrøm, Klaus F. Kofoed, Stephen Schröder, Thomas Zelesny, Matthias Gutberlet, Michael Woinke, Pál Maurovich-Horvat, Béla Merkely, Patrick Donnelly, Peter Ball, Jonathan D. Dodd, Mark Hensey, Bruno Loi, Luca Saba, Marco Francone, Massimo Mancone, Marina Berzina, Andrejs Erglis, Audrone Vaitiekiene, Laura Zajanckauskiene, Tomasz Harań, Malgorzata Ilnicka Suckiel, Rita Faria, Vasco Gama-Ribeiro, Imre Benedek, Ioana Rodean, Filip Adjić, Nada Čemerlić Adjić, José Rodriguez-Palomares, Bruno Garcia del Blanco, Katriona Brooksbank, Damien Collison, Gershan Davis, Erica Thwaite, Juhani Knuuti, Antti Saraste, Cezary Kępka, Mariusz Kruk, Theodora Benedek, Mihaela Ratiu, Aleksandar N. Neskovic, Radosav Vidakovic, Ignacio Diez, Iñigo Lecumberri, Michael Fisher, Balasz Ruzsics, William Hollingworth, Iñaki Gutiérrez-Ibarluzea, Marc Dewey, Jacqueline Müller-Nordhorn

**Affiliations:** 1 Institute of Public Health, Charité – Universitätsmedizin Berlin, corporate member of Freie Universität Berlin, Humboldt-Universität zu Berlin, and Berlin Institute of Health, Berlin, Germany; 2 Institute of Biometry and Clinical Epidemiology and Berlin Institute of Health, Charité – Universitätsmedizin Berlin, corporate member of Freie Universität Berlin, Humboldt-Universität zu Berlin, and Berlin Institute of Health, Berlin, Germany; 3 Department of Radiology, Charité – Universitätsmedizin Berlin, corporate member of Freie Universität Berlin, Humboldt-Universität zu Berlin, and Berlin Institute of Health, Berlin, Germany; 4 Department of Cardiology and Angiology, Charité – Universitätsmedizin Berlin, corporate member of Freie Universität Berlin, Humboldt-Universität zu Berlin, and Berlin Institute of Health, Berlin, Germany; 5 Department of Radiology, Innsbruck Medical University, Innsbruck, Austria; 6 Department of Internal Medicine III, Cardiology, Innsbruck Medical University, Innsbruck, Austria; 7 Department of Imaging Methods, Motol University Hospital, Prague, Czech Republic; 8 Department of Cardiology, Motol University Hospital, Prague, Czech Republic; 9 Department of Cardiology, Rigshospitalet, University of Copenhagen, Copenhagen, Denmark; 10 Department of Cardiology, ALB FILS KLINIKEN GmbH, Goeppingen, Germany; 11 Department of Radiology, ALB FILS KLINIKEN GmbH, Goeppingen, Germany; 12 Department of Radiology, University of Leipzig Heart Centre, Leipzig, Germany; 13 Department of Cardiology, University of Leipzig Heart Centre, Leipzig, Germany; 14 Heart and Vascular Center, Semmelweis University, Budapest, Hungary; 15 Department of Cardiology, Southeastern Health and Social Care Trust, Belfast, UK; 16 Department of Radiology, Southeastern Health and Social Care Trust, Belfast, UK; 17 Department of Radiology, St. Vincent’s University Hospital, Dublin, Ireland; 18 Department of Cardiology, St. Vincent’s University Hospital, Dublin, Ireland; 19 Department of Cardiology, Azienda Ospedaliera Brotzu, Cagliari, CA Italy; 20 Department of Radiology, University of Cagliari, Cagliari, CA Italy; 21 Department of Radiological, Pathological and Oncological Sciences, Sapienza University of Rome, Rome, Italy; 22 Department of Cardiovascular, Respiratory, Nephrology, Anesthesiology and Geriatric Science, Sapienza University of Rome, Rome, Italy; 23 Department of Cardiology, Paul Stradins Clinical University Hospital, Riga, Latvia; 24 Department of Cardiology, Hospital of Lithuanian University of Health Sciences Kaunas Clinics, Kaunas, Lithuania; 25 Department of Radiology, Wojewodzki Szpital Specjalistyczny We Wroclawiu, Wroclaw, Poland; 26 Department of Cardiology, Wojewodzki Szpital Specjalistyczny We Wroclawiu, Wroclaw, Poland; 27 Department of Cardiology, Centro Hospitalar de Vila Nova de Gaia/ Espinho, Vila Nova de Gaia, Portugal; 28 Center of Advanced Research in Multimodality Cardiac Imaging, Cardio Med Medical Center, Tirgu Mures, Romania; 29 Department of Internal Medicine, University of Medicine and Pharmacy, Tirgu Mures, Romania; 30 Department of Cardiology, Institute for Cardiovascular Diseases of Vojvodina, Novi Sad, Sremska Kamenica, Serbia; 31 Faculty of medicine, University of Novi Sad, Novi Sad, Serbia; 32 Department of Cardiology, Hospital Universitari Vall d’Hebron, Institut de Recerca (VHIR), Universitat Autònoma de Barcelona, Barcelona, Spain; 33 BHF Centre of Research Excellence, Glasgow University, Glasgow, UK; 34 Institute of Cardiovascular & Medical Sciences, University of Glasgow, Glasgow, UK; 35 Golden Jubilee National Hospital, Clydebank, UK; 36 Cardiovascular Medicine, University of Central Lancashire, Preston, UK; 37 Department of Cardiology, Aintree University Hospital, Liverpool, UK; 38 Department of Radiology, Aintree University Hospital, Liverpool, UK; 39 Turku PET Centre, Turku University Hospital and University of Turku, Turku, Finland; 40 Heart Center, Turku University Hospital and University of Turku, Turku, Finland; 41 The National Institute of Cardiology, Warsaw, Poland; 42 County Clinical Emergency Hospital, Tirgu Mures, Romania; 43 Department of Radiology and Medical Imaging, University of Medicine and Pharmacy, Tirgu Mures, Romania; 44 Clinic of Internal medicine/Interventional cardiology, Clinical Hospital Center Zemun-Belgrade, Belgrade, Serbia; 45 Faculty of Medicine, University of Belgrade, Belgrade, Serbia; 46 Department of non-invasive diagnostics, Cardiology Division, Clinical Hospital Center Zemun-Belgrade, Belgrade, Serbia; 47 Department of Cardiology, Basurto Hospital, Bilbao, Spain; 48 Department of Radiology, Basurto Hospital, Bilbao, Spain; 49 Department of Cardiology, Royal Liverpool University Hospital, Liverpool, UK; 50 Institute for Cardiovascular Medicine and Science, Liverpool Heart and Chest Hospital, Liverpool, UK; 51 Population Health Sciences, Bristol Medical School, University of Bristol, Bristol, UK; 52 Osteba, Basque Office for Health Technology Assessment, Ministry for Health, Basque Country, Spain

**Keywords:** Chest pain, Angina, Coronary artery disease, Computed tomography angiography, Invasive coronary angiography, Health-related quality of life

## Abstract

**Background:**

Health-related quality of life (HRQoL) is impaired in patients with stable angina but patients often present with other forms of chest pain. The aim of this study was to compare the pre-diagnostic HRQoL in patients with suspected coronary artery disease (CAD) according to angina type, gender, and presence of obstructive CAD.

**Methods:**

From the pilot study for the European DISCHARGE trial, we analysed data from 24 sites including 1263 patients (45.9% women, 61.1 ± 11.3 years) who were clinically referred for invasive coronary angiography (ICA; 617 patients) or coronary computed tomography angiography (CTA; 646 patients). Prior to the procedures, patients completed HRQoL questionnaires: the Short Form (SF)-12v2, the EuroQoL (EQ-5D-3 L) and the Hospital Anxiety and Depression Scale.

**Results:**

Fifty-five percent of ICA and 35% of CTA patients had typical angina, 23 and 33% had atypical angina, 18 and 28% had non-anginal chest discomfort and 5 and 5% had other chest discomfort, respectively. Patients with typical angina had the poorest physical functioning compared to the other angina groups (SF-12 physical component score; 41.2 ± 8.8, 43.3 ± 9.1, 46.2 ± 9.0, 46.4 ± 11.4, respectively, all age and gender-adjusted *p* < 0.01), and highest anxiety levels (8.3 ± 4.1, 7.5 ± 4.1, 6.5 ± 4.0, 4.7 ± 4.5, respectively, all adjusted *p* < 0.01). On all other measures, patients with typical or atypical angina had lower HRQoL compared to the two other groups (all adjusted *p* < 0.05). HRQoL did not differ between patients with and without obstructive CAD while women had worse HRQoL compared with men, irrespective of age and angina type.

**Conclusions:**

Prior to a diagnostic procedure for stable chest pain, HRQoL is associated with chest pain characteristics, but not with obstructive CAD, and is significantly lower in women.

**Trial registration:**

Clinicaltrials.gov, NCT02400229.

## Background

About 20 million patients present annually in Europe with recent onset stable chest pain [1]. Patients with stable chest pain are frequently referred for diagnostic evaluation for coronary artery disease (CAD). A high percentage of patients referred for invasive coronary angiography (ICA) have no obstructive CAD, [2, 3] but even with mild CAD or normal coronary arteries, patients are still at increased risk for future major adverse cardiovascular events and all-cause mortality [4, 5]. After the exclusion of obstructive CAD, many patients continue to experience cardiac symptoms such as palpitations, dyspnoea and chest pain, which negatively impact their health-related quality of life (HRQoL) [6]. HRQoL refers to how people perceive the impact of health conditions or symptoms on their quality of life, in the domains of physical, emotional and social well-being as well as functional capacities in everyday life.

Compared to the general population of adults without CAD, HRQoL is decreased in adults with CAD, with the most pronounced differences in self-rated physical functioning and self-rated overall health [7]. Patients with stable angina pectoris also have lower HRQoL in comparison to the general population [8, 9].

However, not all patients referred for a cardiac diagnostic procedure have typical angina pectoris; atypical angina as well as non-anginal chest discomfort may also prompt a referral for diagnostic evaluation of CAD [3, 10–12]. To our knowledge, no study to date has assessed how these different presentations of chest pain or discomfort relate to HRQoL.

In the present study, we analyse whether there is a relationship between different presentations of chest discomfort and HRQoL in patients referred for diagnostic ICA or computed tomography (CTA). We further test whether pre-diagnostic HRQoL differs between patients with and without obstructive CAD, and male and female patients.

## Methods

### Design, participants and procedure

This study is part of the pilot study of the multicenter DISCHARGE trial (www.dischargetrial.eu; EC-GA 603266), a pragmatic randomized controlled trial at 25 European clinical sites in 16 countries which assesses the comparative effectiveness of ICA and CTA in chest pain patients with a low-to-intermediate likelihood for CAD [13]. One of the purposes of the pilot study was to test the logistic feasibility of a pre-diagnostic assessment of several validated HRQoL measures. The DISCHARGE pilot study was non-randomised and the protocol requested that each site include at least 60 patients with suspected CAD who were routinely referred for diagnostic evaluation (30 CTA and 30 ICA). Referred patients had to have stable chest pain and be at least 30 years of age. Exclusion criteria were not having a sinus rhythm, being pregnant or depending on haemodialysis.

Patients were approached for study participation in the clinics upon their scheduled visit for ICA or CTA. Dependent on local requirements for data acquisition, written and/or oral informed consent was given by all patients participating in the DISCHARGE pilot study. A study nurse interviewed patients to assess the criteria for angina classification. Patients then completed the HRQoL measures on paper. After that, they underwent their scheduled diagnostic procedure in routine care.

All data was stored anonymously. The study was approved by the ethics committee of the Charité- Universitätsmedizin Berlin (EA1/294/13) and by the German Federal Office for Radiation Protection (Z5–2246/2–2014-001).

### Measures

#### Health-related quality of life

##### Short form (SF)-12v2

The SF-12v2 is a generic measure of health status which encompasses an eight-scale profile of functional health and well-being (physical functioning, physical health-related role limitations, bodily pain, general health, vitality, social functioning, emotional health-related role limitations and mental health) [14]. The eight domains are aggregated in a physical component summary score (PCS) and mental component summary score (MCS). Scores were transformed to t-scores [14]. We applied the standard scoring algorithm (based on the SF-12v2 2009 U.S. general population normative sample) because country-specific algorithms are only available for some, but not all countries in DISCHARGE.

##### EuroQoL (EQ-5D-3 L)

The EQ-5D-3 L [15] assesses current HRQoL in five dimensions: mobility, self-care, usual activities, pain/discomfort, and anxiety/depression, each of which can take one of three responses (no problems/some or moderate problems/extreme problems).

We calculated the percentages of patient groups with some/moderate or extreme problems in each domain. Further, health states can be presented along the five dimensions, e.g. health state 11212 represents a patient who indicates some problems (=2) on the usual activities and anxiety/depression dimensions and no problems (=1) on the other dimensions. These health states can be converted to a single index value using available EQ-5D-3 L value sets. We used the value set appropriate for all European countries (study type VAS) [15].

Additionally, the EuroQol includes a visual analogue scale (VAS) on which participants rate how good or bad their own health is today, on a scale from 0 (worst imaginable health state) to 100 (best imaginable health state).

##### Hospital anxiety and depression scale

The HADS assesses the presence and severity of symptoms of anxiety and depression and was originally developed as a screening tool for affective disorders in medical patients [16]. The depression and anxiety subscales each contain seven questions with scores from 0 to 3. Total scores for each subscale thus range from 0 to 21, with higher scores indicating worse anxiety / depression.

#### Angina classification and diagnosis of obstructive coronary artery disease

A study nurse interviewed patients to assess the specific nature of their angina. They were then classified according to one of four angina categories [11]: 1) typical angina pectoris (fulfilling the three criteria a) retrosternal chest discomfort, b) chest discomfort precipitated by exertion, and c) prompt relief [30s – 10 min] of chest discomfort by rest or nitroglycerin) 2) atypical angina pectoris (two out of three criteria for typical angina pectoris), 3) non-anginal chest discomfort (one out of three criteria for typical angina pectoris) and 4) other chest discomfort (none of the criteria for typical angina pectoris).

Obstructive CAD was defined as the presence of at least one coronary artery > 50% diameter stenosis diagnosed by either CTA or ICA. For cases in whom an ICA was performed after CTA, the ICA result was selected for final diagnosis.

### Statistical analyses

Two-level analyses were performed with angina classification, diagnostic outcome (positive versus negative for CAD) and gender as independent factors and age as covariate. The upper level of the analysis consisted of the participating sites, the lower level of the individual patients. Linear random intercept models were used to account for possible correlations arising from the two-level structure. Missing values were imputed using Rubin’s method of multiple imputation with m = 20 samples (imputation method: predictive mean matching). Since only between 1.1 and 3.3% of the values were missing we calculated means, standard deviations and errors from the original sample and used multiple imputation only for the confirmatory part (*P*-values) of the analysis. Intra class correlation coefficients for all measures ranged between 0.05 and 0.08 indicating that site effects are small. The two-sided level of significance was α = 0.05. Since no primary and secondary endpoints were defined for the pilot study, we did not adjust the level of significance for multiple testing.

For graphic display of all measures in one diagram, measures were standardized such that the minimum observation of all measures corresponds to 0 and the maximum observation to 100.

All statistical analyses were performed using the statistical programming language *R* version 3.3.1. For the two-level and multiple imputation analyses the functions *lme* and *mice* from the R packages *nlme* version 3.1–131 and *mice* version 2.46.0, respectively, were used.

## Results

### Characteristics of the study sample

**Fig. 1:**
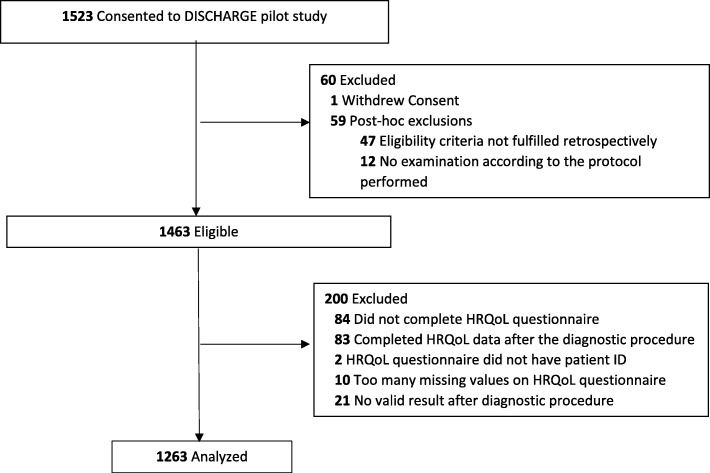
Patient flow-chart

Between April 2014 and July 2017, 25 sites included a total of 1523 patients. Of these, 60 patients were excluded post-hoc and 200 patients had to be excluded from the present analyses due to insufficient or missing HRQoL data or inconclusive / invalid diagnostic result (Fig. 1). Characteristics of the remaining analysis sample from 24 sites (*n* = 1263) are displayed in Table 1. Of these, 617 underwent ICA, 570 had a CT only and 76 had a CT and a subsequent ICA. About 45% of patients had typical angina pectoris, 28% atypical angina pectoris, 23% non-anginal chest discomfort, and 5% other chest discomfort. Overall, 69% of patients were found to not have obstructive CAD (no obstructive CAD was found in 56%, 77% 82% and 83% among patients with typical angina pectoris, atypical angina pectoris, non-anginal and other chest pain, respectively).

**Table 1:** Characteristics of the Study Sample

	Total Sample
	(*n* = 1263)
Age, years	61.1 ± 11.3
Men	683 (54.1%)
Angina Classification	
Typical Angina Pectoris	569 (45.1%)
Atypical Angina Pectoris	352 (27.9%)
Non-anginal chest discomfort	284 (22.5%)
Other chest discomfort	58 (4.6%)
Obstructive CAD based upon results of diagnostic procedure	389 (30.8%)

Values are presented as mean ± SD or *n* (%)

*CAD* coronary artery disease

### Health-related quality of life, angina classification and diagnostic outcome

**Fig. 2:**
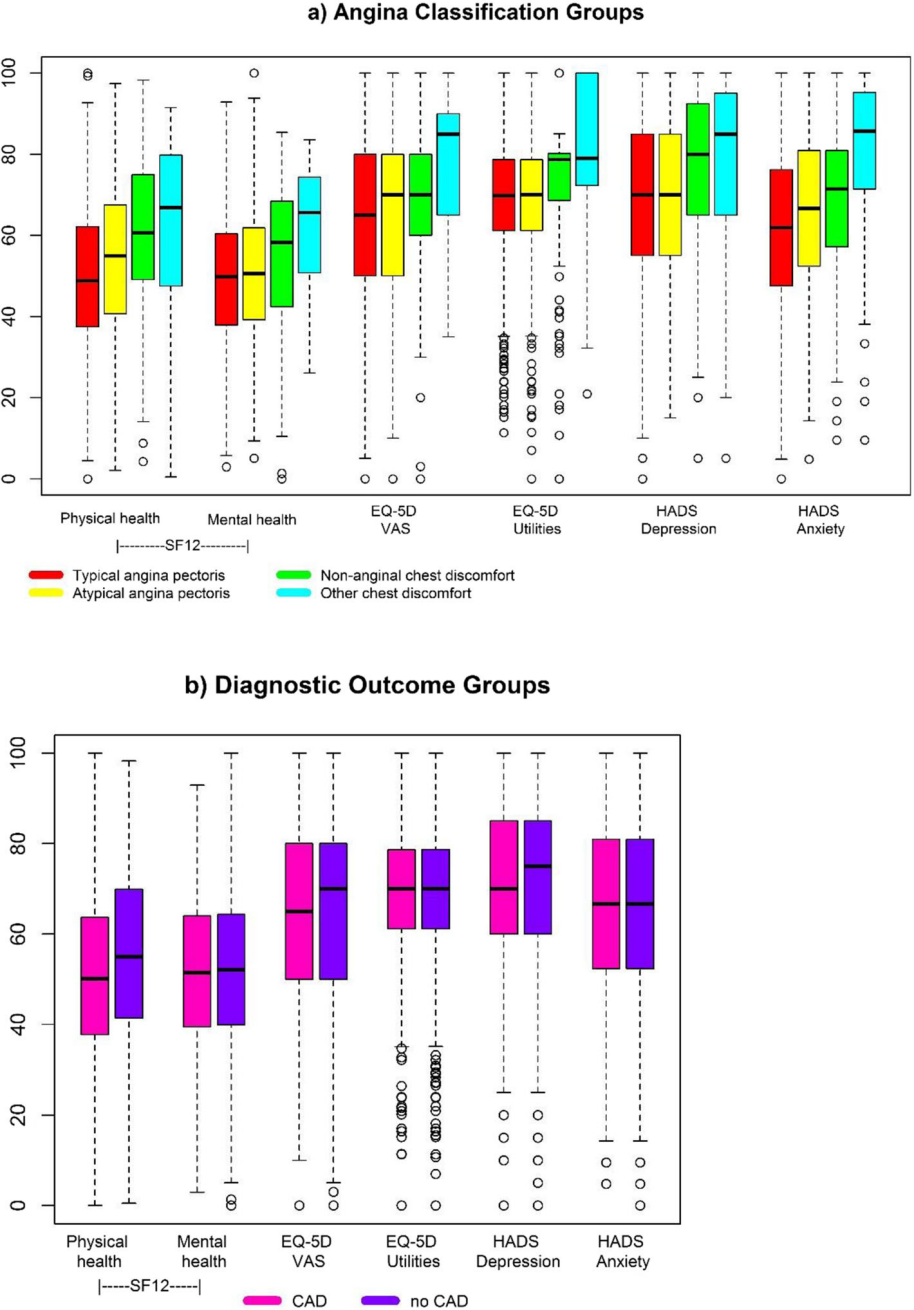
Health-related quality of life in (**a**) angina groups and (**b**) patients with and without CAD. For graphic display, measures were standardized such that the minimum observation of all measures corresponds to 0 and the maximum observation to 100. CAD, Coronary artery disease; HADS, Hospital anxiety and depression scale; VAS, visual analogue scale

Figure 2 shows (standardized) differences in HRQoL measures by angina group and by CAD group. For the Figure, scores for the EurQol and the HADS were recoded so that higher values indicate higher HRQoL on all measures. The mean scores and results of pairwise comparisons (adjusted for age and gender) are displayed in the respective Table 2**.** Patients with typical angina had lower SF-12 physical functioning scores and higher HADS anxiety levels compared to the three other angina groups. On all other measures, patients with typical or atypical angina were similar and had lower scores compared to the two other groups. Patients with non-anginal and other chest discomfort did not differ in their HRQoL on any measure.

**Table 2:** Health-related quality of life measures

	Total sample	Gender			Angina Classification					Diagnostic Outcome		
		Men	Women	*p*	Typical Angina Pectoris	Atypical Angina Pectoris	Non-anginal chest discomfort	Other chest discomfort	p^*^	ObstructiveCAD	No obstructive CAD	*p*
SF-12 Physical Health	43.1 (9.3)	44.2 (9.2)	41.8 (9.3)	0.001	41.2 (8.8)^abc^	43.3 (9.1)^ade^	46.2 (9.0)^bd^	46.4 (11.4)^ce^	< 0.001	41.9 (8.9)	43.7 (9.4)	0.072
SF-12 Mental Health	45.7 (9.8)	47.0 (9.8)	44.1 (9.7)	< 0.001	44.5 (9.5)^ab^	45.1 (9.9)^cd^	47.6 (10.1)^ace^	51.4 (8.7)^bde^	< 0.001	45.6 (10.0)	45.7 (9.8)	0.117
EQ-5D-3 L Visual Analogue Scale	66.3 (18.8)	67.7 (18.3)	64.5 (19.3)	0.010	64.0 (18.1)^ab^	65.6 (19.4)^cd^	69.6 (18.8)^ace^	76.6 (16.8)^bde^	< 0.001	64.1 (18.3)	67.2 (19.0)	0.111
EQ-5D-3 L Utility Score	0.69 (0.21)	0.71 (0.20)	0.66 (0.21)	< 0.001	0.65 (0.21)^ab^	0.68 (0.20)^cd^	0.74 (0.20)^ac^	0.79 (0.22)^bd^	< 0.001	0.67 (0.20)	0.69 (0.21)	0.230
HADS Depression	5.9 (3.95)	5.5 (3.8)	6.4 (4.1)	< 0.001	6.5 (4.0)^ab^	6.1 (3.7)^cd^	4.8 (3.8)^ac^	4.6 (4.5)^bd^	< 0.001	6.0 (4.0)	5.8 (4.0)	0.380
HADS Anxiety	7.5 (4.21)	6.9 (4.0)	8.2 (4.3)	< 0.001	8.3 (4.1)^a^	7.5 (4.1)^a^	6.5 (4.0)^a^	4.6 (4.5)^a^	< 0.001	7.3 (4.1)	7.6 (4.2)	0.861

Unadjusted values are presented as mean ± SD. p-values are based upon multiple imputation analyses and adjusted for age and gender where appropriate. Possible site effects were accounted for by mixed model analysis

* Pairwise comparisons: angina classification groups with a common superscript differ significantly (*p* < 0.05). After applying a Bonferroni correction, some of the comparisons between patients with other or non-anginal chest discomfort and atypical angina pectoris do not reach statistical significance

Measures (possible range in parenthesis): SF-12 Physical and Mental Health (0–100), EQ-5D-3 L Visual Analogue Scale (0–100), EQ-5D-3 L Utility Score (−0.0734–1) and HADS Depression and Anxiety (0–21)

Fourty-eight % of the entire sample (55%, 50%, 38%, 18% among typical angina, atypical angina, non-anginal chest discomfort and other chest discomfort patients) had a HADS anxiety score of > = 8, commonly used as a cut-off for clinical anxiety. Because the anxiety levels were overall very high, we further tested whether patients differed in their anxiety according to the original diagnostic procedure they had been scheduled for. Mean (SD) anxiety scores were slightly higher in patients scheduled for ICA (7.7 ± 4.2) in comparison to those of patients scheduled for CT (7.4 ± 4.3; age and gender- adjusted *p* < 0.01). Importantly, controlling for the scheduled diagnostic procedure did not attenuate the anxiety differences between the chest pain groups which all remained significant.

With and without adjustment for age and gender, there were no significant HRQoL differences between patients with and without obstructive CAD. Only a non-significant trend towards a lower SF-12 physical functioning score was observed for patients with CAD (Table 2).

**Fig. 3:**
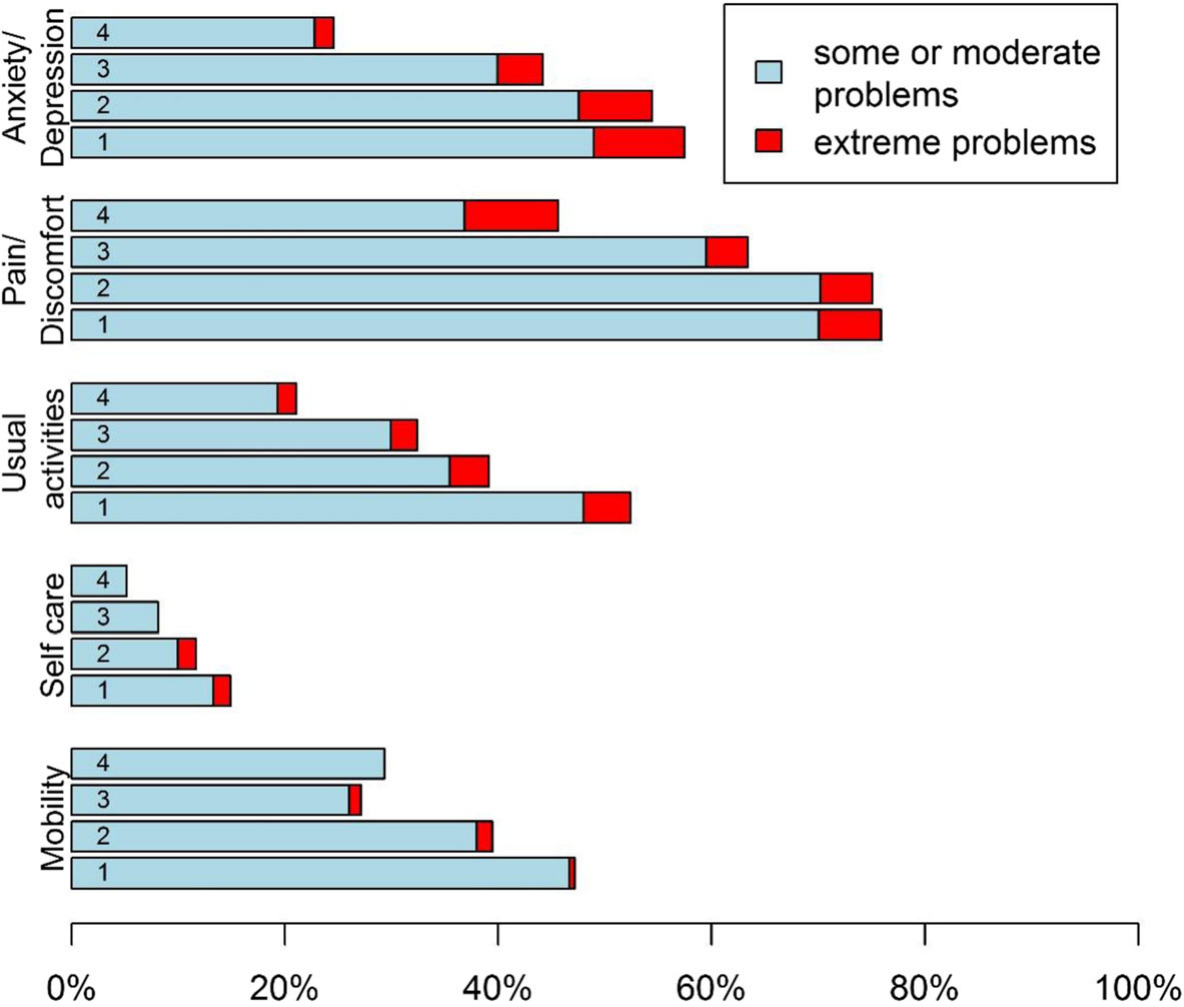
Percentage of patients with some or moderate / extreme problems in EuroQol problem dimensions. Problem dimensions from the EuroQol (EQ-5D-3 L). Groups refer to: 1 = Typical angina pectoris, 2 = Atypical angina pectoris, 3 = Non-anginal chest discomfort and 4 = Other chest discomfort

Figure 3 depicts the percentage of patients with some or moderate / extreme problems in mobility, self-care, usual activities, pain/discomfort, and anxiety/depression according to the EQ-5D-3 L. Again, patients with and without a diagnosis of CAD did not differ on any of these dimensions.

### Gender differences in health-related quality of life

**Fig. 4:**
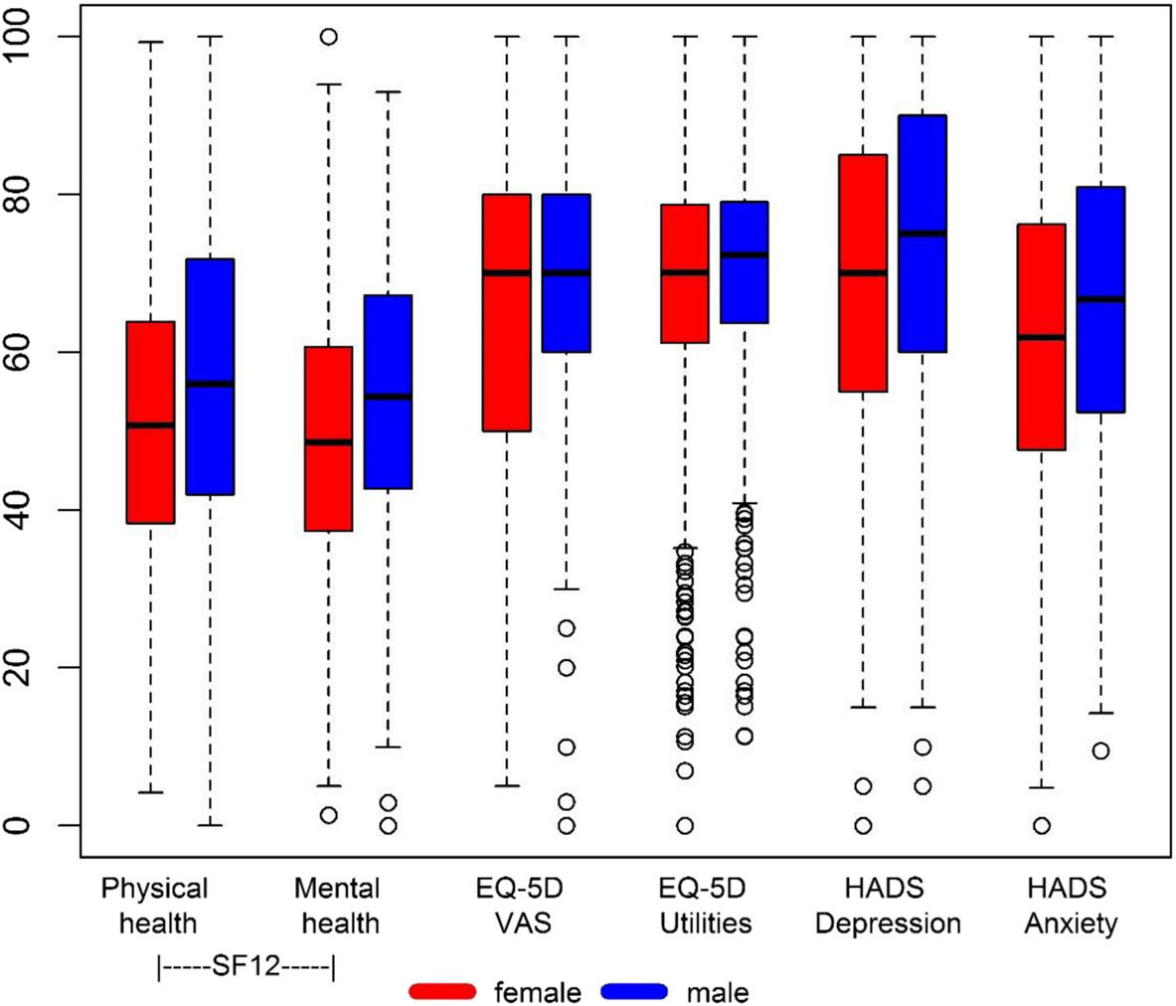
Summary of health-related quality of life differences between women and men. For graphic display, measures were standardized such that the minimum observation of all measures corresponds to 0 and the maximum observation to 100. HADS, Hospital anxiety and depression scale; VAS, visual analogue scale

All measures indicate significantly poorer HRQoL for women (see Fig. 4). Mean (SD) values for the SF-12 physical component summary score and the SF-12 mental component summary score were 41.8 (9.3) and 44.1 (9.7) for women and 44.2 (9.2) and 47.0 (9.8) for men (*p* = 0.001 and *p* < 0.001). For EQ-5D utilities, the mean (SD) score was 0.66 (0.21) for women and 0.71 (0.20) for men (*p* < 0.001). On the VAS, the mean score was 64.5 (19.3) for women and 67.7 (19.3) for men (*p* = 0.010). Mean HADS depression and anxiety scores were 6.39 (4.06) and 8.23 (4.32) for women and 5.47 (3.81) and 6.91 (4.03) for men (both *p* < 0.001). After adjusting for age and angina classification, all gender differences remained significant (all *p* < 0.01).

### Sensitivity analysis

Analyses comparing patients with and without obstructive CAD were repeated in the subsample of patients who had typical angina pectoris. In this subsample, there were also no differences in HRQoL between patients with and without an eventual diagnosis of CAD (all *p*-values > 0.3, data not shown).

## Discussion

This study included 1263 patients from 24 European sites which were referred for elective ICA or CTA.

There were significant differences in pre-diagnostic HRQoL according to patients’ chest pain characteristics. Adjusting for age and gender, patients with typical and atypical angina pectoris had worse quality of life in comparison to patients with non-anginal chest pain and other chest discomfort. These differences were observed across the different functional and emotional aspects of HRQoL and also across the different measures we employed here, confirming their good discriminative validity for different presentations of chest pain.

In comparison to patients with atypical angina, typical angina patients had worse self-rated physical functioning and higher levels of anxiety, but no HRQoL differences between these two angina groups were observed on the other measures. Thus, the SF-12 physical functioning and the HADS anxiety score appear to have a somewhat greater discriminatory power compared to the EuroQol utility score, which is a multidimensional conglomerate of several physical and emotional states.

Minimal clinically important differences (MCID) in these measures have not been assessed for patients with chest discomfort. Conventionally, for the SF-12, a 2-point difference is often assumed a MCID, and differences between typical angina patients and the other chest pain groups exceeded this difference in both the mental and physical health component score of the SF-12.

Comparisons of the HRQoL scores in the present sample with other patient samples should be made with caution due to sample differences in age, gender and clinical characteristics.

Patients in our sample had similar (<= 1 point difference) pre-diagnostic physical functioning and mental health component scores on the SF-12 compared to a sample of 4146 patients with stable angina from the Scottish COmputed Tomography of the HEART (SCOT-HEART) trial, which compared standard care for angina patients with standard care plus CTA [17]. However, SCOT-HEART did not differentiate between different chest pain presentations and also included angina patients with known CAD. In the Cresent trial, [18] which compared the effectiveness and safety of cardiac CTA and functional testing in 350 patients (> 50% with typical chest pain), overall health ratings with the EuroQol VAS were comparable to the ones in our sample. In the recent Prospective Multicenter Imaging Study for Evaluation of Chest Pain (PROMISE) trial, [19] which randomized patients to either standard functional testing or CTA, the pre-diagnostic EuroQol VAS scores were higher in comparison to our sample. However, only 11% of patients included in the PROMISE trial had typical angina pectoris.

In comparison to the HRQoL of 8745 stable coronary heart disease patients with clinical events > 6 months ago in the Euroaspire study, [20] the total sample of the DISCHARGE pilot study patients had similar EuroQol utility and VAS scores, similar HADS depression scores, but substantially lower SF-12 mental health scores (4 point difference) and higher anxiety (1.6 point difference). In all these comparisons, HRQoL scores for the subgroups of patients with typical angina pectoris in our study fall substantially below the HRQoL of the other samples, indicating a high burden associated with this type of chest pain.

Notably, almost half of our entire sample had a HADS anxiety score > =8, the suggested cut-off [16] for probable clinical anxiety. Heightened anxiety prior to a diagnostic procedure has been described before [21] and could be attributed to the uncertainty of the origin of the chest pain / discomfort and the fear associated with heart disease.

Patients with and those without obstructive CAD did not differ in their pre-diagnostic HRQoL, not even in patients with typical angina pectoris. Thus, underlying obstructive cardiac disease cannot explain the HRQoL differences between angina groups. This is in accordance with findings from a Canadian study with 1009 patients referred for elective coronary angiography [22]. Compared with population norms, patients with and without CAD had high anxiety levels and lower HRQoL; the two groups did not differ on most HRQoL measures with the exception of lower self-rated physical functioning in patients with CAD. However, no information on the initial chest pain characteristics were given in the Canadian sample and the CAD outcomes included mild CAD which we did not assess in our study.

There were substantial gender differences in HRQoL: women had consistently worse quality of life on all measures. Gender differences in HRQoL are well documented across a variety of chronic diseases. Women are at greater risk for having clinical and subthreshold depression and anxiety and perceive greater physical and social limitations in their daily lives. In a study with 123 patients with stable CAD and angina pectoris, men and women described their chest pain in a similar manner, but women reported significantly higher physical limitations due to angina pain [23].

It should be noted that HRQoL is also a prognostic factor in cardiac disease. In CAD patients, perceived physical limitations in everyday life were associated with increased mortality and admission for acute coronary syndrome across one year [24]. Depression and anxiety are also prognostic factors for adverse cardiac outcomes and mortality in patients with established CAD [25, 26] and also in patients with stable angina [27, 28]. Whether patients’ HRQoL prior to or after receiving a diagnosis of CAD has an impact on the subsequent treatment recommendations by physicians (e.g., conservative treatment vs. invasive procedures) remains to be studied.

### Strengths and limitations

In this multisite, multinational study, the range of patients with different presentation of chest pain reflects the clinical reality of diagnostic referrals. The pre-intervention assessments ensured that the HRQoL assessment was not influenced by patients’ knowledge of their actual diagnosis. One limitation was that only obstructive CAD was diagnosed. There may well have been HRQoL differences between patients with no CAD, mild (non-obstructive) CAD and obstructive CAD. We did not assess severity and frequency of chest discomfort, which may account for some of the HRQoL differences between the chest pain groups. The EuroQol utility values, for example, have been shown to covary with clinically important differences in angina severity, angina frequency and exertion [29, 30]. We did not include healthy controls from each of the sites. However, the main focus was to analyse whether different presentations of chest pain were associated with HRQoL and all measures were able to discriminate between chest pain groups.

## Conclusions

More than half of the stable chest pain patients referred for diagnostic evaluation did not have typical angina pectoris. Our study showed that differences in chest pain presentation are associated with significant difference in HRQoL. Typical angina patients reported worse physical functioning and higher anxiety compared to patients with atypical, non-anginal or other chest discomfort, to a clinically meaningful extent. Patients with typical or atypical angina significantly differed from the two other groups in anxiety and depression, overall physical functioning, mobility, overall pain and daily functioning. Overall, anxiety levels were close to or above a clinical cut-off. Women consistently reported substantially worse HRQoL compared to men. Notably, patients with versus those without obstructive CAD did not differ in their HRQoL, suggesting that the association between symptoms and HRQoL is driven by symptoms and not by cardiac disease. Further studies should assess how diagnostic evaluation for CAD impacts the HRQoL in patients with different presentations of chest pain, with and without confirmed CAD.

## Data Availability

The datasets used and/or analysed during the current study are available from the corresponding author on reasonable request.

## Abbreviations

CAD: Coronary artery disease
CTA: Computed tomography angiography
HADS: Hospital Anxiety and Depression Scale
HRQoL: Health- related quality of life
ICA: Invasive coronary angiography
MCID: Minimal clinically important difference
MCS: Mental component summary score
PCS: Physical component summary score
PROMISE: Prospective Multicenter Imaging Study for Evaluation of Chest Pain
SCOT-HEART: Scottish COmputed Tomography of the HEART
VAS: Visual analogue scale
