# Correction to: Health-related qualify of life, angina type and coronary artery disease in patients with stable chest pain

**DOI:** 10.1186/s12955-020-01443-8

**Published:** 2020-06-29

**Authors:** Nina Rieckmann, Konrad Neumann, Sarah Feger, Paolo Ibes, Adriane Napp, Daniel Preuß, Henryk Dreger, Gudrun Feuchtner, Fabian Plank, Vojtěch Suchánek, Josef Veselka, Thomas Engstrøm, Klaus F. Kofoed, Stephen Schröder, Thomas Zelesny, Matthias Gutberlet, Michael Woinke, Pál Maurovich-Horvat, Béla Merkely, Patrick Donnelly, Peter Ball, Jonathan D. Dodd, Mark Hensey, Bruno Loi, Luca Saba, Marco Francone, Massimo Mancone, Marina Berzina, Andrejs Erglis, Audrone Vaitiekiene, Laura Zajanckauskiene, Tomasz Harań, Malgorzata Ilnicka Suckiel, Rita Faria, Vasco Gama-Ribeiro, Imre Benedek, Ioana Rodean, Filip Adjić, Nada Čemerlić Adjić, José Rodriguez-Palomares, Bruno Garcia del Blanco, Katriona Brooksbank, Damien Collison, Gershan Davis, Erica Thwaite, Juhani Knuuti, Antti Saraste, Cezary Kępka, Mariusz Kruk, Theodora Benedek, Mihaela Ratiu, Aleksandar N. Neskovic, Radosav Vidakovic, Ignacio Diez, Iñigo Lecumberri, Michael Fisher, Balazs Ruzsics, William Hollingworth, Iñaki Gutiérrez-Ibarluzea, Marc Dewey, Jacqueline Müller-Nordhorn

**Affiliations:** 1 Institute of Public Health, Charité–Universitätsmedizin Berlin, corporatemember of Freie Universität Berlin, Humboldt-Universität zu Berlin, and BerlinInstitute of Health, Berlin, Germany; 2 Institute of Biometry and Clinical Epidemiology and Berlin Institute of Health, Charité–Universitätsmedizin Berlin, corporate member of Freie Universität Berlin, Humboldt-Universität zu Berlin, and Berlin Institute of Health, Berlin, Germany; 3 Department of Radiology, Charité–Universitätsmedizin Berlin, corporate member of FreieUniversität Berlin, Humboldt-Universität zu Berlin, and Berlin Institute ofHealth, Berlin, Germany; 4 Department of Cardiology and Angiology, Charité–Universitätsmedizin Berlin, corporate member of Freie Universität Berlin, Humboldt-Universität zu Berlin, and Berlin Institute of Health, Berlin, Germany; 5 Department of Radiology, Innsbruck Medical University, Innsbruck, Austria; 6 Department of Internal Medicine III, Cardiology, Innsbruck Medical University, Innsbruck, Austria; 7 Department of Imaging Methods, Motol University Hospital, Prague, Czech Republic; 8 Department of Cardiology, Motol University Hospital, Prague, Czech Republic; 9 Department of Cardiology, Rigshospitalet, University of Copenhagen, Copenhagen, Denmark; 10 Department of Cardiology, ALB FILS KLINIKEN GmbH, Goeppingen, Germany; 11 Department of Radiology, ALB FILS KLINIKEN GmbH, Goeppingen, Germany; 12 Department of Radiology, University of Leipzig Heart Centre, Leipzig, Germany; 13 Department of Cardiology, University of Leipzig Heart Centre, Leipzig, Germany; 14 Heart and Vascular Center, Semmelweis University, Budapest, Hungary; 15 Department of Cardiology, Southeastern Health and Social Care Trust, Belfast, UK; 16 Department of Radiology, Southeastern Health and Social Care Trust, Belfast, UK; 17 Department of Radiology, St. Vincent’s University Hospital, Dublin, Ireland; 18 Department of Cardiology, St. Vincent’s University Hospital, Dublin, Ireland; 19 Department of Cardiology, Azienda Ospedaliera Brotzu, Cagliari, CA Italy; 20 Department of Radiology, University of Cagliari, Cagliari, CA Italy; 21 Department of Radiological, Pathological and Oncological Sciences, Sapienza University of Rome, Rome, Italy; 22 Department of Cardiovascular, Respiratory, Nephrology, Anesthesiology and Geriatric Science, Sapienza University of Rome, Rome, Italy; 23 Department of Cardiology, Paul Stradins Clinical University Hospital, Riga, Latvia; 24 Department of Cardiology, Hospital of Lithuanian University of Health Sciences Kaunas Clinics, Kaunas, Lithuania; 25 Department of Radiology, Wojewodzki Szpital Specjalistyczny WeWroclawiu, Wroclaw, Poland; 26 Department of Cardiology, WojewodzkiSzpital Specjalistyczny We Wroclawiu, Wroclaw, Poland; 27 Department of Cardiology, Centro Hospitalar de Vila Nova de Gaia/ Espinho, Vila Nova de Gaia, Portugal; 28 Center of Advanced Research in Multimodality Cardiac Imaging, Cardio Med Medical Center, Tirgu Mures, Romania; 29 Department of Internal Medicine, University of Medicine and Pharmacy, Tirgu Mures, Romania; 30 Department of Cardiology, Institute for Cardiovascular Diseases of Vojvodina, Novi Sad, Sremska Kamenica, Serbia; 31 Faculty of medicine, University of Novi Sad, Novi Sad, Serbia; 32 Department of Cardiology, Hospital Universitari Vall d’Hebron, Institut de Recerca (VHIR), Universitat Autònoma de Barcelona, Barcelona, Spain; 33 BHF Centre of Research Excellence, Glasgow University, Glasgow, UK; 34 Institute of Cardiovascular &Medical Sciences, University of Glasgow, Glasgow, UK; 35 Golden Jubilee National Hospital, Clydebank, UK; 36 Cardiovascular Medicine, University of Central Lancashire, Preston, UK; 37 Department of Cardiology, Aintree University Hospital, Liverpool, UK; 38 Department of Radiology, Aintree University Hospital, Liverpool, UK; 39 Turku PET Centre, Turku University Hospital and University of Turku, Turku, Finland; 40 Heart Center, Turku University Hospital and University of Turku, Turku, Finland; 41 The National Institute of Cardiology, Warsaw, Poland; 42 County Clinical Emergency Hospital, Tirgu Mures, Romania; 43 Department of Radiology and Medical Imaging, University of Medicine and Pharmacy, Tirgu Mures, Romania; 44 Clinic of Internal medicine/Interventional cardiology, Clinical Hospital Center Zemun-Belgrade, Belgrade, Serbia; 45 Faculty of Medicine, University of Belgrade, Belgrade, Serbia; 46 Department of non-invasive diagnostics, Cardiology Division, Clinical Hospital Center Zemun-Belgrade, Belgrade, Serbia; 47 Department of Cardiology, Basurto Hospital, Bilbao, Spain; 48 Department of Radiology, Basurto Hospital, Bilbao, Spain; 49 Department of Cardiology, Royal Liverpool University Hospital, Liverpool, UK; 50 Institute for Cardiovascular Medicine and Science, Liverpool Heart and Chest Hospital, Liverpool, UK; 51 Population Health Sciences, Bristol Medical School, University of Bristol, Bristol, UK; 52 Osteba, Basque Office for Health Technology Assessment, Ministry for Health, Basque Country, Spain

**Correction to: Health Qual Life Outcomes 18, 140 (2020)**

**https://doi.org/10.1186/s12955-020-01312-4**

The original article [1] contained an error in co-author, Balazs Ruzsics’s name which has since been corrected.
